# Risk of latent tuberculosis infection in children living in households with tuberculosis patients: a cross sectional survey in remote northern Lao People's Democratic Republic

**DOI:** 10.1186/1471-2334-9-96

**Published:** 2009-06-17

**Authors:** Tuan H Nguyen, Peter Odermatt, Gunther Slesak, Hubert Barennes

**Affiliations:** 1 Institut de la Francophonie pour la Médecine Tropicale, Vientiane, Lao PDR; 2 Swiss Tropical Institute, Department Public Health and Epidemiology, PO Box, 4002 Basel, Switzerland; 3 Service Fraternel d'Entraide, P.O. Box 56, Luang Nam Tha Provincial Hospital, Luang Namtha, Lao PDR

## Abstract

**Background:**

Tuberculosis is highly prevalent in Laos (289 per 100,000). We evaluated the risk of latent tuberculosis infection (LTBI) among children (0–15 years) living with tuberculosis patients in rural northern Laos.

**Methods:**

In a cross sectional survey of 30 randomly selected villages, 72 tuberculosis patients were traced and their 317 contacts (148 were children) investigated using a questionnaire, a tuberculin skin tests (positive: > = 10 mm), a 3-day sputum examination for acid-fast bacilli (AFB), and chest radiography.

**Results:**

None of the 148 contact-children received prophylaxis, one had cervical tuberculosis; the risk for LTBI was 31.0%. Awareness of the infectiousness of tuberculosis was low among patients (31%) and their contacts (31%), and risky behavior was common. After multivariate logistic analysis, increased LTBI was found in children with contact with sputum positive adults (OR: 3.3, 95% CI: 1.4–7.7), patients highly positive sputum prior to treatment (AFB >2+; OR: 4.7, 95% CI: 1.7–12.3), and living in ethnic minorities (OR: 5.4, 95% CI: 2.2–13.6).

**Conclusion:**

The study supports the importance of contact tracing in remote settings with high TB prevalence. Suggestions to improve the children's detection rate, the use of existing guidelines, chemoprophylaxis of contact-children and the available interventions in Laos are discussed. Improving education and awareness of the infectiousness of TB in patients is urgently needed to reduce TB transmission.

## Background

Asia is estimated to have 55% of the 9.27 million worldwide incident cases of TB of which 78% are actually detected [1]. In resource-poor areas with a high prevalence of TB, children carry a large proportion of the overall burden. Almost one million cases of childhood TB are estimated each year; 10 to 20% are fatal [2,3]. Despite its importance, childhood TB remains a neglected disease [4].

Investigation of people exposed to patients with infectious TB (contact tracing) is the key to TB control in countries with low TB incidence. In high incidence countries contact tracing is uncommon and debated [5].

Contact tracing is generally accorded a low priority by National Tuberculosis Programs (NTP) of low-income countries. The reasons are partly because of the workload imposed by active cases, who have a higher priority, partly because of the lack of standardized diagnostic criteria for latent tuberculosis infection (LTBI), and because treatment of LTBI is usually not provided except for children under five years of age [5].

In the Lao People's Democratic Republic (Laos) TB incidence and prevalence are estimated at 151 and 289 per 100,000 in 2007, respectively [6]. The annual risk of TB infection (ARTI) was estimated at 1.2% [7]. The TB detection rate has increased from 26% in 2000 to 44% in 2006 [6].

The diagnosis relies mainly on sputum examination for acid-fast bacilli (AFB) and chest radiograph (CXR) [8]. Neither method is available in remote rural areas. For children, sputum samples are difficult to obtain. In addition childhood TB is mainly extra-pulmonary or involves exclusively in intra-thoracic lymph nodes. Less than 20% of sputum specimens obtained from children with pulmonary TB are positive for AFB [9]. Hence, children are rarely enrolled in TB treatment. In 2007 in Laos only 0.6% of all the notified new sputum positive cases were children under 15 years of age [6]. Generally 15–20% of TB patients are expected to be children [4]. In high incidence countries TB disease and LTBI is estimated to be 7% and 40.4%, respectively, among children <15 years living with TB patients [5].

We evaluated the prevalence of LTBI among children living with confirmed TB patients in a remote setting in Luang Nam Tha (LNT) province.

## Methods

### Study area

Laos is a low income country with a per capita income of USD 500 with 39% of the population classified as poor [10]. Directly observed treatment short-course (DOTS) was started in 1995 and was introduced in LNT province in 2004 [4]. To date multi-drug-resistant TB has not been reported from Laos. Tuberculin skin tests (TST) are no longer in use and *Mycobacterium tuberculosis *culture is not available. Regarding HIV, Laos remains a low prevalence country with an estimated 0.08% HIV seroprevalence in the adult population [11].

**Figure 1:**
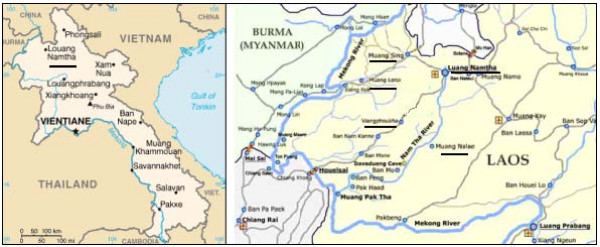
**Map of the study area: Luang Nam Tha Province, North of Laos**.

LNT is located in the North, bordering Myanmar and China (Figure 1). It is a multi-ethnic province with a low population density (16 people/km^2^). The 145,231 inhabitants belong to 3 main ethnic groups. 34 ethnic minorities are present. A total of 24,965 families live in 380 villages (census 2005). There are one provincial and one military hospital in the provincial capital and 5 district hospitals. Access to modern health care is low due to a variety of reasons including infrastructure, cultural differences and financial constraints.

### Study procedure

From January 2004 to February 2006, 219 TB patients (95% pulmonary TB) were diagnosed and treated at the LNT provincial hospital. They were traced back to 74 villages of which 30 villages were randomly selected for the study. This number was chosen due to feasibility reasons and the available time frame allowing an estimated follow-up of 80 to 90 patients and 5 contact persons per patient. The selected villages included 83 TB patients (37.9%) who were all sputum AFB positive. All TB patients and individuals living in the same household (children ≤ 15 years old and adults) were eligible for the study and are referred to as "participants". Adult contacts (age > 15 years) were included in order to account for a possible additional risk of infection for children by exposure to undiagnosed TB disease in other adult contacts.

The study was performed from March to June 2006. Using a pre-tested questionnaire study participants were interviewed for medical history, vaccinations, number of missed days of treatment (for patients), degree (such as eating in the same plate, sleeping together with the patient) and duration of contacts (time from first symptoms to treatment), characteristics of the household and distance to health centers, knowledge of the transmission of the disease and practices related to cough and spitting. Translation into ethnic languages was performed by one of the team investigator (PM).

### Physical examination

A physical examination of the study participants was performed. It included the presence of a BCG vaccination scar in contacts and an investigation for clinical signs of TB patients. The patient's nutritional status was assessed by calculating the body mass index (BMI ≤ weight (kg)/height (m)^2^). Adults were considered underweight if BMI was ≤ 18.5 and severely underweight if BMI < 16. Height was measured to the nearest 0.1 cm using a wooden measuring board. Weight was assessed using an electronic bathroom scale for adults (precision ± 100 g).

### Laboratory procedures

The following procedures were applied to all participants. We collected three sputum samples according to the Lao National Guidelines [7]. On each sample a Ziehl-Neelsen stained slide was done and examined for the presence of AFB at the LNT provincial hospital.

A dose of 0.1 ml tuberculin (Tubertest, Aventis Pasteur SA, France) was injected intradermally in the left forearm [12]. 48–72 hours later, the site of injection was palpated by the same investigator. The largest transverse diameter of the induration was measured with a transparent flexible ruler. TST was classified "positive" if the induration diameter was ≥ 10 mm [12].

Villagers with clinical signs or symptoms of TB were referred to the LNT provincial hospital for further medical examination, chest X-ray and eventually treatment. Patients and their families received counseling about the disease. All participants gave informed witnessed oral consent; in children parents or caretakers had to consent. The study was approved by the Ministry of Health's Ethical Council of Medical Sciences for Health Research. The study was performed in accordance with the Declaration of Helsinki [13].

### Definitions

LTBI was defined as a positive TST in the absence of TB disease [5]. TB disease was suspected if the interviewees reported chronic cough (longer than 3 weeks), weight loss, night sweats, and fever, or if signs of extra-pulmonary TB were observed [8].

We defined a TB patient as compliant if at any time during the treatment course at least 90% of the medicine was taken. The pill count assessment of NTP, double checked with the district hospital was used for the assessment. We defined a TB treatment to be completed if the patient had finished at least 95% of his treatment days according to his treatment category [8]. Degree of contact was defined as close if patients and children usually share the same meal or the same bed, or live in the same room. Duration of contact was the reported time period from the onset of the disease to the beginning of the directly observed therapy (DOT).

### Data management and analysis

Data was entered in EpiData freeware http://www.epidata.dk. All records were cross-checked with the original data sheets. Analysis was carried out with STATA, Versions 8 (Stata Corporation, College Station, TX, USA). Chi-squared and Fisher's exact tests and Student's t-test were used to compare categorical variables and continuous data, respectively. 95% confidence intervals (95% CI) were calculated for continuous and categorical data. The following risk factors were evaluated in an univariable analyses: sex, age, ethnic group, residential area, distance to health centre, family size, number of people sharing the same house, room size, degree and duration of contacts, and compliance to treatment. All factors with a p value ≤ 0.2 were then fitted into a multivariable logistic regression model. We considered p < 0.05 as statistically significant.

## Results

**Figure 2:**
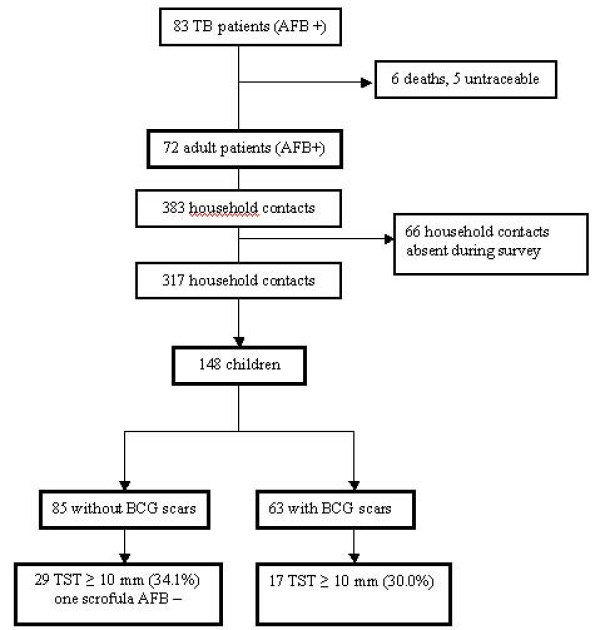
**Flow chart of the investigated TB patients and close-contact children and adults in Luang Nam Tha Province, Northern Laos**.

We followed up at home all 83 index patients from 30 villages in 5 districts who had been treated for AFB sputum positive TB (Figure 1). Six of them had already died (6.2%) and five were untraceable (6.0%). Finally, we enrolled 72 adult patients (86.7%) and their 317 household members: 148 children (50 children ≤ 5 years) and 169 adult close contacts (Figure 2). Appointments were not possible for 66 contacts (17.2%) who mainly were adults and grown up children gone to work.

Interviews were conducted after a mean of 345.8 days (standard deviation ± 212.65) after the start of treatment. The main characteristics of TB patients are shown in Table 1. Patients lived in wooden houses or bamboo huts with one main room. The majority was illiterate. Forty-nine of 72 TB patients (68.1%) did not know how the infection is transmitted. Significantly more patients complained of symptoms among those who stopped treatment compared to those under treatment (61% *vs *56%, p = 0.001). 37 (51.4%) had chronic cough and 1 (13.9%) was still AFB+ at the time of the investigation. Nineteen patients (26.4%) were severely underweight.

**Table 1:** Characteristics of treated tuberculosis patients (n = 72)

	Completed treatment	Ongoing treatment	Total
	n = 42 (%)	N = 30 (%)	n = 72 (%)
Male	29 (69.0)	19 (63.3)	48 (66.7)
Age (years, range)	45.5 (17–75)	43.4 (17–73)	46.6 (16–75)
Belong to ethnic minority	32 (76.1)	20 (66.6)	38 (52.7)
- Lao Thung	13 (30.9)	6 (20.0)	18 (25.0)
- Lao Sung	19 (45.2)	14 (46.6)	25 (34.7)
Main occupation			
- farmers	29 (69.0)	21 (70.0)	50 (69.4)
- no occupation*	11 (26.1)	7 (23.3)	18 (25.0)
Education			
- illiterate	26 (61.9)	20 (66.6)	46 (63.4)
- primary education	10 (23.8)	8 (25.0)	18 (25.0)
- secondary education	6 (14.2)	2 (11.1)	8 (11.1)
Mean distance to Health Centre (km) (range)	5.5 (± 3.6)	4.0 (1–10)	4.7 (3.9–5.5)
Compliant with treatment	32 (75.1)	26 (86.6)	58 (80.5)
- mean duration of treatment before stop (days)	215.4 (57–240)	NA	199 (62–235)
Clinical observations	26 (61.9)	17 (56.6)	43 (59.7)
- chronic cough**	20 (47.6)	17 (56.6)	37 (51.4)
- persistent expectoration***	18 (42.8)	11 (36.6)	29 (40.3)
Weight (kg, range)	44.16 (± 8.0)	40.60 (27–54)	42.7 (22–64)
Underweight: BMI < 18.5	26 (61.9)	20 (66.6)	46 (63.9)
- severely underweight: BMI < 16	9 (21.4)	10 (33.33)	19 (26.4)

Mean with 95% confidence interval

* Others were students (n = 2), civil servants (n = 1), teachers (n = 1), and retired (n = 1)

** Other symptoms were night sweat (n = 11, 6.5%), and fever (n = 7, 4.1%)

*** More than 3 weeks

Patients who completed their treatment were found to have better nutritional status than others (mean BMI 18.1, 95% CI: 17.2–19.0 *vs. *mean BMI 16.9, 95% CI: 16.1–17.8, p = 0.06). The compliance rate was calculated to be 86.6% in patients currently under treatment, and 75.1% in those who had already finished their treatment.

Patients' risky behavior regarding transmission of TB to their close contacts are shown in Table 2. Most of TB patients shared drinks (97.1%) and meals (93.0%) with the family members and 84.7% shared the sleeping place with other members. All patients reported to cough in the house.

**Table 2:** TB transmission relevant characteristics of tuberculosis patients

	Patients
	n = 72 (%)
Haemoptysis before treatment	40 (55.5)
AFB found before treatment	72 (100)
AFB found during follow-up	1 (1.3)
House size (in m^3^, range)	89.0 (77–100)
Number of people living in the same house*	5.3 (4.6–5.9)
Shares sleeping place with other family members	61 (84.7)
Coughs in shared rooms	72 (100)
Spits inside the house	37 (51.3)
Drinks from the same glass	69 (97.1)
Eats together with the other family members	67 (93.0)

* Mean with 95% confidence interval

Of 265 contacts (167 adults and 98 children > = 6 years), 184 (69%, 114 adults and 70 children) did not know how TB was transmitted. Three adult close contacts were found AFB positive (1.8%). They were referred to the hospital for TB treatment.

The main characteristics of contact children are shown in Table 3. One AFB negative child had extra pulmonary TB (1.7%). This child suffered from scrofula but responded well to TB treatment. No contact child had been receiving TB prophylaxis. Significantly more children aged 0–5 years had scars (27/50, 54.4%) from BCG vaccination than those aged 6–15 years (36/98, 36.7%; p = 0.04). The prevalence of LTBI was 31.1% (95% CI: 23.7–39.2) and increased with age from 26.0% (13/50) in children below 5 years to 35.7% (35/98) in children between 6 – 15 years. The difference was not statistically significant. The proportion of positive TST did not differ between those with and without a BCG scar (26.9% (17/63) *vs. *34.1% (29/85), p = 0.3).

**Table 3:** Characteristics of child household contacts of TB patients

	Children
	n = 148 (%)
Male	79 (53.4)
Age (years, range)*	1.8 (0.1–15)
- below 5 years	50 (33.8)
Belong to ethnic minorities	114 (77.0)
Shares sleeping place with TB patient	71 (48.0)
Education	
- illiterate	83 (56.1)
- primary education	50 (33.8)
- secondary education	15 (10.1)
Fever	1 (0.7)
BCG scars	63 (42.6)
Mean diameter of TST induration (mm)*	15.9 (14.8–16.9)
TST ≥ 10 mm	46 (31.1)
Sputum positive for AFB ^£^	0

TST: tuberculin skin test; AFB: acid-fast bacilli

* Mean with 95% confidence interval

^£ ^Sputum obtained from 37 children

Results of the univariable analyses and multivariable logistic regression analysis are showed Table 4 and 5, respectively. The following risk factors for contact children were identified: a highly positive sputum with more than 2+ of AFB prior to treatment (OR 6.2, p < 0.001) and belonging to the Lao Sung ethnic group (OR 3.2, p = 0.03).

**Table 4:** Risk factors for LTBI of Lao children contacts of TB patients (univariable analyses)

	Odds Ratio	Std. Err.	p	95% CI
Patients with complaints	4.30	5.32	0.23	0.38 – 48.68
Lao Sung ethnic group *	4.24	2.18	0.00	1.54 – 11.65
Children with sputum *	3.75	2.83	0.07	0.85 – 16.44
Children below 5 years *	3.68	1.91	0.01	1.32 – 10.22
Illiterate*	3.30	2.62	0.13	0.69 – 15.69
Patients with initial sputum ++ *	2.17	0.84	0.04	1.01 – 4.66
Patient spitting on follow-up*	1.87	0.69	0.09	0.90 – 3.85
Room under 30 m^3^	1.06	0.60	0.91	0.34 – 3.25
Sex	1.04	0.36	0.8	0.52 – 2.09
Number of family members ≥ 7	0.92	0.32	0.81	0.46 – 1.83
Distance to health centre ≥ 1 km	0.88	0.32	0.75	0.43 – 1.83
No knowledge on transmission	0.85	0.34	0.70	0.39 – 1.88
Direct filiation to TB patient	0.76	0.26	0.43	0.38 – 1.50
TB patients with chronic cough	0.74	0.27	0.42	0.36 – 1.52
BCG scars	0.64	0.23	0.22	0.31 – 1.30
No compliance to treatment	0.60	0.26	0.24	0.25 – 1.41

* variable included in the multi-variate analysis

**Table 5:** Risk factors for LTBI of Lao children contacts of TB patients (multivariable analysis)

TB Patients	Odds Ratio	Std. Err.	p	95% CI
initially sputum ++	6.20	3.49	< 0.001	2.05 – 18.70
in Lao Sung ethnic group	3.25	1.80	0.03	1.09 – 9.64
spitting at follow-up	3.09	1.85	0.05	0.95 – 10.01

## Discussion

Our study found a prevalence of LTBI of 26–36% in children living with TB patients in remote settings of Northern Laos. In parallel we revealed a very low awareness of the infectiousness of tuberculosis both in contacts and in TB patients. Risky behavior of TB patients and household contacts was very common. Overall, the patients were illiterate, middle-aged and lived in remote areas. These factors can explain the found poor knowledge regarding TB transmission, the poor compliance and risk behavior since the disease has not been well understood.

Most children with TB are hardly infectious. Therefore, TB control programs in settings with limited resources target with priority older age groups [14]. In addition, the diagnosis of pulmonary TB is particularly difficult in children. This has resulted in TB being still an under-diagnosed and hence neglected disease in children. Although, it causes substantial morbidity and mortality [14]. There is a wide gap between the expected and the detected number of TB cases in children in Laos. In the Lao NTP guideline a special score chart for the diagnosis of childhood TB is provided [8]. However, in practice in children who can not produce sputum an attempt to diagnose TB is rarely performed, and thus TB infection remains untreated.

### Risk of latent TB infection

Despite some limitations, i.e. 17.2% of contact could not be traced for interviews, our study documents that a high proportion of children living in households with TB patients have a LTBI (31.0%). This finding underlines the crucial importance of contact investigation: i) in filling the gap between expected and diagnosed number of child TB cases; ii) identifying high-priority candidates for treatment of LTBI in Laos [5]. Unlike adults who have a 10–15% chance of developing the disease in their lifetime, contact children are at highest risk of developing overt disease. Previous study showed that up to 50% of infants will develop the disease within 3–9 months of infection and 25% of children 1–5 years of age; 15% of adolescents will develop the disease within 1–2 years of infection [15]. Targeting the 0–5 years old age-group has been recommended particularly for those nations that are unable to implement full-scale contact investigation [5].

About 17.2% of contacts were absent during the survey. We assume that these were mainly people gone to their fields who potentially were healthier than those remaining in the village. This might have led to a slightly higher rate of detected TB disease in contacts, but should not influence the rate of LTBI which is per definition without symptoms. Another possible limitation concerns the contact group that might have also included persons with only transient contacts to patients but who were no longer there during our study period.

In our study we did not examine a control group of children in households without TB patients. The previous national survey provided an estimated prevalence of positive TST of 5.8% (range 1.2% to 11.8%, 95% CI: 5.1–6.5) among these children [16]. In reference to this we could roughly estimate that the Lao children of our study living in households with TB patients have a 6-times greater risk of TB infection (assuming a constant ARTI). A study of household children contacts in India showed rates of LTBI of 41% in contacts of adult patients with bacillary disease [17].

The proportion of children with LTBI was lower than reported in other studies in low and middle income countries using a similar cut-off for the TST induration diameter of 10 mm [5]. Rates as high as 47.8%, 55.6% or 69.2% have been reported in Bangkok [18], neighboring Chang Rai [5] and the Philippines [15], respectively. Several factors might be responsible for this lower rate in our study: i) the lower population density; ii) the lower HIV prevalence; and iii) the high rate of malnutrition. TST has a poor sensitivity for detecting tuberculosis infection especially in children with HIV co-infection [19]. HIV seroprevalence was not investigated but is still very low in Laos [11]. In a 2006 prevalence study in 4 ethnic minority villages in LNT all 924 villagers were HIV negative (G. Slesak, unpublished data). Due to the frequent association of TB with HIV a lower HIV rate may cause less casual contact transmission of TB and thereby might have lowered the proportion of children with LTBI in our study. Poor nutritional status has been reported to decrease TST reactivity in children by depressing immune responsiveness to BCG [20]. This on the other hand could lead to an underestimation of the true rate of LTBI. In fact no assessment of children's nutritional status was performed in this study but a prevalence of 74% of severe chronic malnutrition (stunting) in children has been reported from a study in the LNT province [21].

A higher variation of LTBI between age groups is commonly reported [5] and is explained by the limited years of exposure to tuberculosis that children have had compared with adults. In this study the trend was not significant which could be related to the rather small sample size.

Studies have generally shown a grading in the indicators of transmission (active tuberculosis and LTBI) by closeness and duration of contact with the infectious source [5]. Only two main risk factors were found to be significant in our study. Current contact with sputum positive adults was close to significance, (p = 0.05, 95% CI: 0.95–10.01). Current contact with sputum positive adults and patients highly positive sputum prior to treatment, were related to the density and infectivity of the case. Current contact with sputum positive adults was close to significance, (p = 0.05, 95% CI: 0.95–10.01). Current factors such as age, size of the household, sharing the same bed, number of people living in the same house and BCG scars were not significantly associated with the presence of a LTBI. Some of these current factors were described to be confounders after multivariate analysis [20]. Our findings can be explained by the following factors: the specific study site where household characteristics tended to be very similar in these remote low income areas, the limited study size, and the lack of control households.

BCG scars were not significantly associated with transmission. Despite vaccination with BCG, a positive TST in a child who has had close contact with an infected adult is assumed to most likely represent infection with *M. tuberculosis*. Treatment of this latent infection should be considered, especially if the child is younger than 5 years [22].

### Contact tracing

Contact tracing in high incidence countries is generally given a low priority. A recent review suggests that this strategy merits revision. Contact tracing is a means to improve early case detection, prevent further propagation of drug resistance and is a cost-effective method for early identification of secondary cases to decrease transmission of *M. tuberculosis *in high-incidence areas [5]. Contact tracing contributes to meet the case-detection target rates of the NTP in Laos. In our field study we diagnosed 4 new TB patients (1.2%) among contacts, one of whom was a child. The 3 previously undiagnosed but sputum-positive adults have undoubtedly contributed to the maintenance of TB transmission and further increased the risk of infection for contact children.

### Preventive chemoprophylaxis

Contact tracing also facilitates preventive chemoprophylaxis for children in close contact with a TB patient. A significant reduction of contacts' progression to TB disease with Isoniazid chemoprophylaxis has been reported [23]. Children with a significant reaction should be treated with Isoniazid if they do not have active TB [8,23]. This deserves further attention in our setting since no child at high risk of disease received any prophylaxis. This shows a possible poor knowledge of the national recommendations described in the National TB policy. According to that children below 5 years of age without TB disease but in contact with a sputum positive patient should be given preventive therapy. Isoniazid is recommended for 6 months followed by a BCG vaccination in the unvaccinated child [7]. Other reasons are that TST are usually not available in Laos and the lack of further techniques to diagnose TB disease in children.

In similar resource limited settings where chest X-rays and TST are not readily available it certainly would be helpful to follow the suggested algorithms of TB contact management for children [24]. According to that children below 5 years of age without TB disease but in contact with a sputum positive patient should be given preventive therapy.

### Improving case detection

Despite its limitations, the tuberculin skin test is able to predict progression to active disease in all risk groups, and there is evidence of the efficacy of preventive treatment for individuals with a positive skin test who are at risk of progression. Making TST available in Laos will help trained health workers make decisions. Improving case detection by other diagnostic techniques such as gastric lavage or nasopharyngeal aspiration and sputum induction, or strings tests are limited by the requirement of overnight fasting, repeated specimens, attendance at the clinic and similarly restrained by the need for culture of the specimen [25,26]. To improve the diagnosis of TB disease in children in developing countries ideally there should be a simple rapid and non-invasive test that could be done also at health centre level. There is no diagnostic gold standard for the presence of LTBI. Serological tests have not been found useful [9]. Tests based on interferon expression in response to *M. tuberculosis *antigens are more sensitive than TST but require techniques and skills that are rarely available in remote areas; they are also expensive (≈US $20/test)[27]. Meanwhile the high rate of TB in Laos could be reduced by: improving education and awareness of health staff and population, systematic contact investigation of all index patients using mobile teams to improve active household case detection, contacts surveillance and patient compliance, and thereby improving the correct implementation of the existing national TB guidelines.

### Awareness about risk of infection

Awareness of the infectiousness of TB was low (30%) in case-patients, adults and child contacts. This raises questions about the quality of the given counseling and health education during DOTS at the concerned hospitals. This low level of awareness might explain the persistence of risky behavior such as indiscriminate spitting and close contacts while coughing or sleeping. Both, low awareness and risky behavior, might have contributed to the high proportion of LTBI in contact children.

## Conclusion

Children in Laos living in remote areas in contact with TB patients are at high risk of LTBI and, hence, active disease. Our study provides further arguments that contact tracing for TB control in developing countries is beneficial. Increasing awareness of the infectiousness of TB within health staff, patients and the general population is urgently needed. Contact tracing and chemo-prophylaxis should be applied to reduce the risk of LTBI children to develop active TB. Future research must focus on suitable rapid field tests for children. Despite the difficulties experienced in resource-limited countries, the management of childhood TB infection could be vastly improved by better implementation of existing guidelines and available interventions.

## List of abbreviations

(LTBI): Latent tuberculosis infection; (AFB): acid-fast bacilli microscopy; (TB): tuberculosis; (OR): odd-ratio; (95% CI): 95% confidence interval; (NTP): National Tuberculosis Program; (Laos): Lao People's Democratic Republic; (DOTS): directly observed treatment strategy; (TST): tuberculin skin tests; (LNT): Luang Nam Tha Province; (BMI): body mass index

## Competing interests

The authors declare that they have no competing interests.

## Authors' contributions

HTN, PO, HB designed the study. HTN, MP, GS, HB conducted the field work. HB and HTN performed the statistical analysis and interpretation of the data together with PO. HB wrote the manuscript with support from PO, HTN, GS and MP. All authors read and approved the final version of the manuscript.

## Pre-publication history

The pre-publication history for this paper can be accessed here:

http://www.biomedcentral.com/1471-2334/9/96/prepub
